# Patterns and prevalence of depression, tobacco, and caffeine use among university students from different majors in Lebanon: a cross-sectional study

**DOI:** 10.3389/fpubh.2025.1605086

**Published:** 2025-06-04

**Authors:** Afife El Hashem, Angela Bou Saba, Anna Maria Bou Madi, Caroline Mahfouz, Clara Derian, Elie El Husseiny, Jeanne-Marie Hanna, Maguy El Hayek, Shafika Assaad

**Affiliations:** Fundamental Health Sciences Department, School of Medicine and Medical Sciences, Holy Spirit University of Kaslik, Jounieh, Lebanon

**Keywords:** depression, tobacco consumption, caffeine use, university students, Lebanon

## Abstract

**Background:**

Mental health plays a fundamental role in overall wellbeing, with depression being one of the most common mental health disorders worldwide. University students face significant academic, financial, and social pressures, making them particularly vulnerable to depression. Research showed difference in consumption of psychoactive substances such as caffeine and tobacco among genders and academic fields. It suggests also a link between the use of these substances and an increase in depressive symptoms. The consumption of these substances among all university students in Lebanon from multiple majors and the relationship between this intake and mental health remains underexplored.

**Materials and methods:**

This cross-sectional study surveyed university students across Lebanon to assess their tobacco and caffeine consumption and its relationship with depression. A self-administered online questionnaire, including the Beck Depression Inventory, was used to measure depression levels and collect data on substance use patterns. A total of 626 convenient responses were analyzed, incorporating both descriptive and inferential statistical analyses.

**Results:**

The study found that 31.15% of university students experienced some level of depression. Gender differences were observed in tobacco and caffeine consumption: cigarette smoking was significantly higher among males (25%) than females (7.4%), while no significant gender-based differences were found for electronic cigarette and shisha use. Among caffeinated products, chocolate was the most consumed (92.9% males, 98.1% females), followed by other types of coffee (71.9% males, 80.5% females). Significant gender differences were noted in Arabic coffee, chocolate, tea, and cigarette consumption, but not for energy drinks, e-cigarettes, or waterpipes. Depression levels did not significantly differ between genders for most substances, except for cigarette smoking, where female smokers exhibited significantly higher depression scores than male smokers (*p* = 0.032). Additionally, depression levels varied across academic majors, with architecture students displaying the highest levels and medical students the lowest. Tobacco consumption is higher among non-medical majors, whereas caffeine is higher among health and non-medical majors.

**Conclusion:**

This study highlights the need for targeted mental health interventions, awareness campaigns, and accessible psychological support services for university students. Future research should explore additional sociocultural factors influencing substance use and mental health.

## 1 Introduction

Mental health is a fundamental aspect of wellbeing, influencing emotions, cognition, and daily behaviors. Depression is one of the most prevalent mental health disorders globally, affecting over 280 million individuals and significantly impacting academic, social, and occupational functioning (1). University students are particularly vulnerable due to academic pressures, financial constraints, and social challenges (2).

The link between depression and the consumption of psychoactive substances, such as tobacco and caffeine, has been widely studied. Research suggests that the increased use of these substances correlates with heightened depressive symptoms (2–4). In Lebanon, tobacco products including cigarettes, waterpipes, and e-cigarettes are easily accessible, reflecting trends observed in other Mediterranean countries (2, 3, 5, 6). Similarly, caffeine consumption, primarily through coffee, energy drinks, and tea, is common among university students, especially during intense academic periods (7). While many students consume these substances for cognitive enhancement and alertness (2, 8), excessive intake has been linked to anxiety and depressive symptoms (9, 10). However, some studies have found no significant correlation between caffeine consumption and depression (11).

Consumption of these substances was examined among genders to see if differences exist. Different results were seen in studies. In Medina KSA and in Bahrain, males were found to be caffeine consumers more than females (11, 12). However, in UAE and USA, females were higher caffeine consumers than males (8, 13). Additionally, a study conducted on Palestinian students revealed disparities between different caffeinated products. Females consume chocolate more than males, but males consume energy drinks more than females and no differences were seen in the consumption of coffee and tea (2). Among majors, caffeine consumption varied in certain studies with medical majors consuming less than non-medical (7) but no difference in others (8, 14). Concerning tobacco consumption, contradictory results were also found in literature. In Palestine, male university students consumed tobacco products more than females (2). But in Sweden, females used cigarettes more than males (5). For depression, females in Palestine showed a higher prevalence than males (2) similarly to other countries (15). And among majors, non-medical students were more depressed than medical ones in Palestine (2). It is interesting to find out which of these results will be matched with findings in Lebanon.

Regional studies in the MENA region, such as those conducted in Palestine and Saudi Arabia, have revealed alarming rates of substance use and its association with depression (2, 7, 14). However, it remains unclear whether these findings are applicable to the Lebanese context, where cultural and socioeconomic factors may influence consumption patterns and mental health outcomes (16, 17). Since the country passed lately through several crisis, mental health of students was affected, and nicotine was seen to be consumed by those impacted (17).

In Lebanon, previous research has focused primarily on medical students. A 2019 study found a significant correlation between daily caffeine consumption and stress levels (18). Additionally, a 2018 study reported that 95.6% of medical and pharmacy students consumed caffeine, with intake rising significantly during exams (19). However, no study has comprehensively assessed tobacco and caffeine consumption among the broader university student population in Lebanon or examined their relationship with depression in participants from various academic fields.

Despite the known health risks, tobacco and caffeine consumption remain widespread among Lebanese university students (16, 18). These substances are often used as coping mechanisms for stress and fatigue, potentially exacerbating psychological distress (2, 5).

This study aims to determine the prevalence and usage patterns of tobacco and caffeine among Lebanese university students, to assess the relationship between depression and the consumption of tobacco and caffeine, and to compare depression levels and substance use among genders and academic fields.

## 2 Materials and methods

### 2.1 Design and sample settings

A cross-sectional descriptive study design was conducted during the academic year 2024–2025 to assess the prevalence of tobacco and caffeine consumption and its relationship with depression levels, taking into account gender and academic field. Participants were recruited from various universities across Lebanon, and only students aged 18 years and older were eligible to participate. To ensure data completeness, all survey questions were mandatory, resulting in a dataset without missing values. The study included anonymous 626 participants who completed an online survey. The sample obtained is a convenient sample, where data was collected only from participants who consented and accepted to participate in the study and no incentive was offered.

### 2.2 Questionnaire

The questionnaire was self-administered, multiple-choice, online, and divided into two sections. The first section consisted of 23 questions designed to collect demographic information, including gender, academic field, habits, and reasons for tobacco and caffeine use. It aimed to capture patterns of cigarette smoking, waterpipe use, and e-cigarette consumption, as well as the frequency and motivations behind caffeine intake through coffee, tea, energy drinks, and chocolate. The second section utilized the Beck Depression Inventory (BDI), a validated 21-item scale assessing the severity of depressive symptoms. The BDI classifies depression into six categories: normal (1–10), mild mood disturbance (11–16), borderline clinical depression (17–20), moderate depression (21–30), severe depression (31–40), and extreme depression (>40) (20).

### 2.3 Statistical analysis

The statistical analysis was conducted using IBM SPSS Statistics 27, following a 95% confidence interval (CI) for all inferential tests. A *p*-value of < 0.05 was considered statistically significant. Descriptive statistics were used to summarize categorical variables, while means and standard deviations were calculated for continuous variables. Inferential statistics included independent samples *t*-test for variables with two groups, such as gender. For variables with three or more groups, we applied ANOVA if the homogeneity of variance test (Levene's test) shows *p* > 0.05 (indicating equal variances). If *p* < 0.05, we used the Welch test to account for unequal variances. Additionally, if at least one group has a low sample size, we applied the Kruskal-Wallis test as a non-parametric alternative. The chi-square test was performed to evaluate associations between categorical variables.

### 2.4 Ethical considerations

Ethical approval for this study was granted on December 20, 2024, by the Research Ethics Committee of the Higher Center for Research at Holy Spirit University of Kaslik, under the reference number HCR/EC 2024-082. Participation was voluntary, and informed consent was obtained from all students before completing the survey. Data collection was conducted anonymously to ensure confidentiality, and all responses were used exclusively for research purposes.

## 3 Results

### 3.1 Prevalence and usage patterns of tobacco and caffeine among Lebanese university students

Table 1 illustrates the consumption patterns of tobacco and caffeinated substances among male and female students. Cigarette smoking was significantly more prevalent among males (25%) compared to females (7.4%), whereas the use of waterpipes (26.5% males, 22.3% females) and electronic cigarettes (23.5% males, 17.4% females) did not show significant gender-based differences. Energy drink consumption was slightly higher among males (34.2%) than females (29.5%). Arabic coffee consumption was reported by 47.4% of males and 37.7% of females, while other types of coffee were more commonly consumed by females (80.5%) than males (71.9%). Tea consumption showed a similar pattern, with higher consumption among females (86.3%) compared to males (71.9%). Chocolate was the most frequently consumed caffeine source, with slightly higher intake among females (98.1%) than males (92.9%).

**Table 1:** Consumption patterns of tobacco and caffeinated substances by gender.

**Substance**	**Category**	**Males *n* (%)**	**Females *n* (%)**	***p*-Value (chi-square)**
Cigarettes	Yes	49 (25.0)	32 (7.4)	0.000
	No	147 (75.0)	398 (92.6)	
Waterpipes (shisha)	Yes	52 (26.5)	96 (22.3)	0.251
	No	144 (73.5)	334 (77.7)	
Electronic cigarettes	Yes	46 (23.5)	75 (17.4)	0.077
	No	150 (76.5)	355 (82.6)	
Energy drinks	Yes	67 (34.2)	127 (29.5)	0.243
	No	129 (65.8)	303 (70.5)	
Arabic coffee	Yes	93 (47.4)	162 (37.7)	0.021
	No	103 (52.6)	268 (62.3)	
Other types of coffee	Yes	141 (71.9)	346 (80.5)	0.017
	No	55 (28.1)	84 (19.5)	
Tea	Yes	141 (71.9)	371 (86.3)	0.000
	No	55 (28.1)	59 (13.7)	
Chocolate	Yes	182 (92.9)	422 (98.1)	0.001
	No	14 (7.1)	8 (1.9)	

Table 2 examines the percentages of male and female university students who consume different caffeinated substances and their respective depression levels. The chi-square test for differences in caffeinated substances consumption revealed significant gender differences in the consumption of Arabic coffee (*p* = 0.021), other types of coffee (*p* = 0.017), chocolate (*p* = 0.001), and tea (*p* = 0.000), suggesting that gender plays a role in caffeine intake patterns.

**Table 2:** Percentages of male and female university students who consume different caffeinated substances and their respective depression levels.

**Caffeinated substances**	**Gender (caffeinated substances = yes)**	***p*-Value (chi-square) for consumption differences**	**Depression level**	***p*-Value (*t*-test) for depression differences**
Arabic coffee	Males: 47.4%	0.021	8.84	0.978
	Females: 37.7%		8.81	
Energy drinks	Males: 34.2%	0.243	8.16	0.160
	Females: 29.5%		9.75	
Other types of coffee	Males: 71.9%	0.017	8.63	0.554
	Females: 80.5%		9.12	
Chocolate	Males: 92.9%	0.001	8.24	0.519
	Females: 98.1%		8.70	
Tea	Males: 71.9%	0.000	8.09	0.398
	Females: 86.3%		8.76	

Table 3 examines the percentages of male and female university students who use different tobacco substances and their respective depression levels. The chi-square test for differences in tobacco substances consumption found a significant association between cigarette smoking and gender (*p* = 0.000), indicating that males were more likely to smoke cigarettes than females. However, no significant gender-based differences were found for electronic cigarette (*p* = 0.077) or waterpipe use (*p* = 0.251).

**Table 3:** Percentages of male and female university students who use different tobacco substances and their respective depression levels.

**Tobacco substances**	**Gender (tobacco substances = yes)**	***p*-Value (chi-square) for consumption differences**	**Depression level**	***p*-Value (*t*-test) for depression differences**
Cigarettes	Males: 25%	0.000	9.63	0.032
	Females: 7.4%		14.25	
Electronic cigarettes	Males: 23.5%	0.077	9.41	0.265
	Females: 17.4%		11.36	
Shisha	Males: 26.5%	0.251	9.62	0.699
	Females: 22.3%		10.19	

Table 4 details the frequency of tobacco and caffeine consumption by gender. Among cigarette smokers, the majority of both males (73.5%) and females (71.9%) consumed fewer than one pack per day, while heavy smoking (more than one pack per day) was uncommon (4.1% males, 3.1% females). Waterpipe usage was predominantly occasional, with 61.5% of males and 63.5% of females reporting infrequent use, whereas daily use was higher among males (19.2%) compared to females (10.4%). Daily electronic cigarette consumption was significantly higher among males (69.6%) than females (41.3%). For energy drink consumption, the percentages among genders were similar however, a difference was observed in the frequencies of use with occasional consumption being the highest, followed by weekly and then daily. Among caffeine consumers, daily Arabic coffee consumption was reported by 43% of males and 37% of females, while other coffee types were consumed daily by 51.1% of males and 55.2% of females. Tea and chocolate consumption showed a higher frequency among females, with 24.5% consuming tea daily compared to 19.9% of males, and 46.4% consuming chocolate daily compared to 43.4% of males.

**Table 4:** Frequency of psychoactive substance consumption by gender.

**Substance**	**Frequency category**	**Males *n* (%)**	**Females *n* (%)**	***p*-Value (chi-square)**
Cigarettes per day	Less than one pack	36 (73.5)	23 (71.9)	0.947
	One pack	11 (22.4)	8 (25.0)	
	More than one pack	2 (4.1)	1 (3.1)	
Waterpipe (shisha)	Occasionally	32 (61.5)	61 (63.5)	0.270
	Weekly	10 (19.2)	25 (26.0)	
	Daily	10 (19.2)	10 (10.4)	
Electronic cigarettes	Occasionally	9 (19.6)	35 (46.7)	0.006
	Weekly	5 (10.9)	9 (12.0)	
	Daily	32 (69.6)	31 (41.3)	
Energy drinks	Occasionally	49 (73.1)	96 (75.6)	0.931
	Weekly	15 (22.4)	26 (20.5)	
	Daily	3 (4.5)	5 (3.9)	
Arabic coffee	Occasionally	38 (40.9)	78 (48.1)	0.524
	Weekly	15 (16.1)	24 (14.8)	
	Daily	40 (43.0)	60 (37.0)	
Other types of coffee	Occasionally	41 (29.1)	89 (25.7)	0.680
	Weekly	28 (19.9)	66 (19.1)	
	Daily	72 (51.1)	191 (55.2)	
Tea	Occasionally	80 (56.7)	147 (39.6)	0.002
	Weekly	33 (23.4)	133 (35.8)	
	Daily	28 (19.9)	91 (24.5)	
Chocolate	Occasionally	36 (19.8)	68 (16.1)	0.531
	Weekly	67 (36.8)	158 (37.4)	
	Daily	79 (43.4)	196 (46.4)	

Table 5 highlights the primary motivations for tobacco and caffeine consumption among students. Males most commonly developed the habit of smoking over time (36.1%), while curiosity was the leading reason for females (32.0%). Stress relief was a common reason for both groups (24.1% males, 24.2% females). For caffeine consumption, habitual use over time was the most frequently reported reason (51.6% males, 46.5% females). Functional motivations varied, with males more likely to consume caffeine to wake up in the morning (17.2%) and females to stay awake throughout the day (19.3%).

**Table 5:** Motivations for tobacco and caffeine consumption by gender.

**Motivation**	**Males *n* (%)**	**Females *n* (%)**	***p*-Value (chi-square)**
**Motivation to start using tobacco**			
Curiosity	19 (22.9)	41 (32.0)	0.472
Habit formed over time	30 (36.1)	40 (31.3)	
Peer influence	14 (16.9)	16 (12.5)	
Stress relief	20 (24.1)	31 (24.2)	
**Motivation for caffeine consumption**			
Habit formed over time	99 (51.6)	200 (46.5)	0.081
To wake up in the morning	33 (17.2)	59 (13.7)	
To relieve discomfort (headache, irritation)	7 (3.6)	42 (9.8)	
To stay awake during the day	26 (13.5)	83 (19.3)	
To lose weight	2 (1.0)	4 (0.9)	
To enhance mental performance	18 (9.4)	30 (7.0)	
To enhance physical performance	7 (3.6)	12 (2.8)	

### 3.2 Assessment of the relationship between depression and consumption of tobacco and caffeine among Lebanese university students

**Figure 1:**
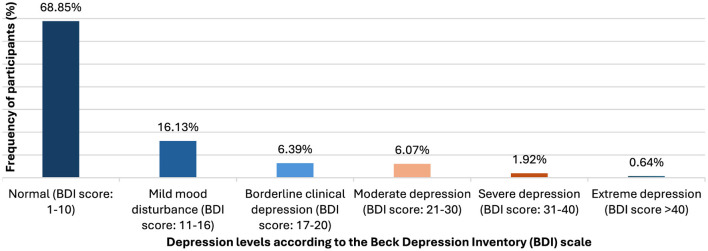
Level of depression among Lebanese university students.

The overall prevalence of depression among Lebanese university students was found to be 31.15%, as illustrated in Figure 1, which presents the distribution of depression severity among participants. The majority of students (68.85%) fell within the normal range, while 16.13% exhibited mild mood disturbances. The remaining 14.02% displayed moderate to extreme levels of depression. The mean depression score among participants was 8.61, with a standard deviation of 8.14, suggesting significant variability in reported depressive symptoms.

In Table 2 that assesses depression with caffeinated substance consumption, independent samples *t*-tests showed no significant differences in depression scores between male and female consumers of caffeinated substances (*p* > 0.05) despite significance in consumption of caffeinated substance.

In Table 3 that assesses depression with tobacco substance consumption, *t*-test comparing depression scores among cigarette smokers found that female smokers had significantly higher depression levels than male smokers (*p* = 0.032), whereas no significant differences were observed for e-cigarette (*p* = 0.265) or waterpipe users (*p* = 0.699). In parallel, we also had significant different consumption between males and females for cigarettes but not e-cigarettes and shisha.

### 3.3 Comparison of depression level and substance use among genders and academic fields

Table 6 presents the relationship between sociodemographic factors and depression levels. No statistically significant differences were found in depression scores among different age groups (ANOVA, *p* = 0.26), gender (*t*-test, *p* = 0.426), academic year (Kruskal–Wallis, *p* = 0.563), or work status (*t*-test, *p* = 0.129). However, depression scores differed significantly across academic fields (Welch's test, *p* = 0.042), highlighting potential academic stress variations among students from different disciplines.

**Table 6:** Comparison of depression level among gender, sociodemographic and academic fields.

**Variables**	**Total sample (*N* = 626)**	**Mean total depression level**	**Standard deviation**	***p*-Value**
**Age**				ANOVA
18–20	381 (60.86%)	9.02	8.18	0.26
21–25	218 (34.82%)	8.09	8.18	
26–30	27 (4.31%)	7.15	7.05	
**Gender**				*t*-test
Male	196 (31.31%)	8.23	8.434	0.426
Female	430 (68.69%)	8.79	8.008	
**Current academic year**				Kruskal–Wallis
1st−2nd	227 (36.26%)	8.93	7.77	0.563
3rd−4th	316 (50.48%)	8.28	8.096	
5th−6th	64 (10.22%)	9.45	10.129	
7th +	19 (3.04%)	7.58	5.501	
**Work**				*t*-test
Yes	236 (62.30%)	7.98	7.186	0.129
No	390 (37.70%)	9	8.654	
**Field of study**				Welch test
Medicine	133 (21.25%)	7.73	8.21	0.042
Engineering	105 (16.77%)	8.95	7.9	
Health related sciences	91 (14.54%)	9.18	8.31	
Sciences	90 (14.38%)	8.15	7.2	
Business	79 (12.62%)	8.76	8.88	
Arts and letters	52 (8.31%)	12.56	7.96	
Law	51 (8.15%)	7.18	7.03	
Architecture	25 (3.99%)	11.04	9.89	

Table 7 presents depression levels across different academic majors stratified by gender. The highest depression scores were observed among architecture students (14.10 females, 6.40 males), while the lowest scores were recorded among medical students (7.42 females, 8.45 males). Further analysis showed significant gender differences in depression scores in health sciences (*p* = 0.041), law (*p* = 0.049), architecture (*p* = 0.008), and arts and letters (*p* = 0.021). In all cases, except for arts and letters, females exhibited higher depression levels than males.

**Table 7:** Comparison of depression levels between genders among majors.

**Major**	**Depression level**		***p*-Values (*t*-test)**
	**Males**	**Females**	
Medicine	8.45	7.42	0.509
Health related sciences	6.41	9.53	0.041
Law	5.25	10.38	0.049
Engineering	8.40	7.93	0.738
Business	7.91	9.43	0.454
Architecture	6.40	14.10	0.008
Sciences	7.83	6.87	0.548
Arts and letters	18.38	9.70	0.021

Table 8 compared the consumption of tobacco products between genders among different majors. Significant gender differences were found in cigarette usage among medicine (*p* = 0.001), law (*p* = 0.002), architecture (*p* = 0.003) and science majors (*p* = 0.000), with male students being higher consumers than females. Specifically, law exhibited the highest male cigarette smoking rate (50%), while female cigarette smoking was highest in engineering (18.2%) among all majors, but in law (10.3%) for majors with significant gender differences. However, consumption of waterpipes and electronic cigarettes generally did not show significant gender differences except in medicine (*p* = 0.025), and science (*p* = 0.000) for electronic cigarettes where males also had higher usage. Among all majors, arts and letters students were the highest consumers of waterpipes compared to other majors, for both males (37.5%) and females (36.4%). For electronic cigarettes, architecture students had highest consumption in e-cigarettes among males (40%), but business students among females (31.80%).

**Table 8:** Comparison of tobacco products consumption between genders among majors.

**Majors**	**Genders**	**Tobacco products**					
		**Cigarettes**	***p*** **-Value (chi-square) for cigarettes**	**Waterpipes**	***p*** **-Value (chi-square) for waterpipes**	**Electronic cigarettes**	***p*** **-Value (chi-square) for electronic cigarettes**
Medicine	Males	12.50%	0.001	17.50%	0.723	17.50%	0.025
	Females	0%		15.10%		5.40%	
Health-related sciences	Males	11.80%	0.774	35.30%	0.423	17.60%	0.807
	Females	9.50%		25.70%		20.30%	
Law	Males	50%	0.002	8.30%	0.156	16.70%	0.522
	Females	10.30%		28.20%		25.60%	
Engineering	Males	34%	0.064	36%	0.241	32%	0.458
	Females	18.20%		25.50%		25.50%	
Business	Males	20%	0.288	20%	0.165	14.30%	0.07
	Females	11.40%		34.10%		31.80%	
Architecture	Males	40%	0.003	0%	0.461	40%	0.211
	Females	0%		10%		15%	
Sciences	Males	24.10%	0.000	34.50%	0.002	34.50%	0.000
	Females	0%		8.20%		4.90%	
Arts and letters	Males	37.50%	0.101	37.50%	0.951	12.50%	0.44
	Females	13.60%		36.40%		25%	

Table 9 compared the consumption of caffeinated products between genders among different majors. No significant gender differences were seen in any major for Arabic coffee and energy drinks consumption. Significant gender differences were seen in business (*p* = 0.027) and science (*p* = 0.042) for the consumption of other types of coffee, engineering (*p* = 0.002) and architecture (*p* = 0.035) for tea consumption. For chocolate in arts and letters (*p* = 0.011) and architecture (*p* = 0.041). For all of these significant differences, females consumed more than males. For males, health related sciences students were the highest consumers of other types of coffee (88.20%), energy drinks (52.9%) and tea (82.4%) whereas law students consumed Arabic coffee (75%) and chocolate (100%) more than other majors. For females, business students were the highest consumers of other types of coffee (88.60%) and energy drinks (50%), architecture students consumed Arabic coffee (50%) and chocolate (100%) more than other majors, whereas for tea consumption, engineering students were the highest consumers (89.10%).

**Table 9:** Comparison of caffeinated products consumption between genders among majors.

**Majors**	**Genders**	**Caffeinated products**									
		**Arabic coffee**	***p*** **-Value (chi-square) for Arabic coffee**	**Other types of coffee**	***p*** **-Value (chi-square) for other types of coffee**	**Energy drinks**	***p*** **-Value (chi-square) for energy drinks**	**Tea**	***p*** **-Value (chi-square) for tea**	**Chocolate**	***p*** **-Value (chi-square) for chocolate**
Medicine	Males	45%	0.161	75%	0.868	25%	0.299	80%	0.217	97.50%	0.822
	Females	32.30%		76.30%		17.20%		88.20%		96.80%	
Health-related sciences	Males	47.10%	0.255	88.20%	0.485	52.90%	0.089	82.40%	0.774	94.10%	0.251
	Females	32.40%		81.10%		31.10%		85.10%		98.60%	
Law	Males	75%	0.08	58.30%	0.141	16.70%	0.522	75%	0.445	100%	
	Females	46.20%		79.50%		25.60%		84.60%		100%	
Engineering	Males	46%	0.666	70%	0.338	32%	0.298	64%	0.002	92%	0.137
	Females	41.80%		78.20%		41.80%		89.10%		98.20%	
Business	Males	54.30%	0.237	68.60%	0.027	45.70%	0.705	65.70%	0.102	88.60%	0.097
	Females	40.90%		88.60%		50%		81.80%		97.70%	
Architecture	Males	40%	0.689	80%	1	40%	0.668	40%	0.035	80%	0.041
	Females	50%		80%		30%		85%		100%	
Sciences	Males	37.90%	0.984	69%	0.042	34.50%	0.085	79.30%	0.245	96.60%	0.586
	Females	37.7%		86.90%		18%		88.50%		98.40%	
Arts and letters	Males	37.50%	0.951	75%	1	25%	0.534	75%	0.532	75%	0.011
	Females	36.40%		75%		36.40%		84.10%		97.70%	

## 4 Discussion

### 4.1 Discussion of results

This study assessed the prevalence of depression and its association with tobacco and caffeine use among university students in Lebanon, revealing a depression rate of 31.15%. This finding is comparable to regional data, notably the 33.1% prevalence reported among Palestinian university students (2). However, it is considerably lower than the 54.2% rate identified in another recent Lebanese study confined to a single university setting (17). These variations underscore the impact of diverse institutional and sociocultural contexts on mental health outcomes. In fact, the multiple crises Lebanon has passed through, contributed to the deterioration of students' mental health. Loss of job due to the economic crisis, being impacted by Beirut port explosion and the political unrest were related to burnout, depression and anxiety (17).

Unlike previous studies reporting significant gender differences in depression, particularly highlighting a higher prevalence among females (15, 17), our results found no statistically significant overall gender disparity. This divergence may reflect evolving societal norms and gender roles within the Lebanese context, thus warranting further exploration to understand underlying factors better.

Regarding caffeine use, our findings correspond with a Palestinian study that identified chocolate as the primary source of caffeine, followed by tea and non-Arabic coffee, with higher consumption among females (2). However, these results contrast with studies from the UAE, Saudi Arabia, and Bahrain, where Arabic coffee predominates (7, 8, 12). This indicates the influence of distinct cultural preferences on consumption patterns. Our study demonstrated that for these highly consumed substances, both males and females consume chocolate and other types of coffee daily but tea occasionally. In Palestine, chocolate is mainly consumed as source of caffeine weekly, whereas tea and coffee daily for both males and females (2). Concerning the cause behind the consumption of caffeinated products, our findings show that habit formed over time is the main one for both males and females. The second major cause was waking up in the morning for 17.2% of males and staying awake during the day for 19.3% of females. Similarly, these reasons were reported in studies among Lebanese medical students in 2019 (18), all Lebanese university students in 2016 (16) and other countries (13).

Tobacco consumption patterns observed in our study revealed waterpipes as the most frequently used tobacco product among both genders, aligning with findings from Palestinian universities (2). Compared to the general population in Lebanon in 2021, females consumed similarly more waterpipes, however adult males preferred cigarettes (6) as seen also in KSA among university students (21). Concerning the frequency, our study revealed that waterpipes are consumed occasionally like Palestinian university students (2), and less than one pack of cigarettes is consumed daily by both genders, like in the general Lebanese population in 2021 (6) and university students in Riyadh (22). According to our findings, the causes behind these consumptions were curiosity, habit formed over time or stress relief mainly, which were reported by other studies either as major causes (2) or minor ones (22).

Our study found that 24.1% of males and 24.2% of females use tobacco to relief stress and that there is no significant difference in depression level between genders contrary to other studies where females smoke to cope with stress more than male since they suffer more from anxiety disorders (15, 23, 24).

Our results indicated a significant difference in the consumption of caffeinated products between males and females for all substances except energy drinks. However, no significant gender difference was observed in depression levels associated with any caffeinated substance. This suggests that although caffeine consumption patterns differ by gender, these differences are not significantly related to depression among males and females. These findings are consistent with studies conducted in Palestine and Saudi Arabia, which also reported no significant association between caffeine intake and depression levels by gender (2, 11). Conversely, another study from Saudi Arabia highlighted a positive correlation between stress and higher caffeine consumption (7), and a finding supported by a meta-analysis demonstrated a positive association between stressful life events and depression (25). Additionally, research involving Korean middle school students found that higher caffeine intake was linked to increased depression and insomnia (9). On the other hand, other studies indicated a negative association between low caffeine intake and depression, while high caffeine consumption showed either no significant relationship or a positive association with depression (26, 27).

Our study emphasizes that significant gender differences were evident exclusively in cigarette consumption, where males smoked significantly more than females, whereas no notable gender differences were found in the use of e-cigarettes and shisha. Interestingly, female cigarette smokers exhibited significantly higher depression scores than their male counterparts, while no gender difference was noted for depression levels associated with e-cigarette and shisha use. Therefore, cigarette smoking, although more prevalent among males, appears to have a stronger correlation with increased depression in females. Similar findings were observed in a Palestinian study, which reported an association between cigarette use and depression but not for e-cigarettes or shisha (2). Furthermore, a recent Lebanese study identified nicotine as a contributing factor to depression among university students (17). Likewise, a systematic review by Hu et al. (28) demonstrated consistent associations between depression and both current and former smoking habits. In the United States, previous research indicated internalizing problems, including depression, as significant predictors for initiating tobacco use (3). Consistent with our results, another U.S. study found that vulnerability to depression significantly increased the likelihood of smoking, particularly among female university students (4).

Our findings revealed significant variations in depression levels across different academic fields, with architecture and arts majors reporting the highest depression levels, while sciences and medical students exhibited lower levels. These outcomes align with results from a Palestinian study, which reported a higher depression prevalence among non-medical students (34.3%) compared to medical students (31.7%) (2). Additionally, although not statistically significant, previous Lebanese research indicated that students majoring in arts and business had higher proportions of moderate to severe depression and anxiety symptoms compared to their medical counterparts (17). Several factors have been documented to affect the mental health of architecture students, including stringent deadlines, demanding workloads, and rigorous scheduling (29). Furthermore, studies from the USA revealed significant depression rates in arts and humanities students, unlike those in science, technology, engineering, and mathematics (STEM) fields (30). When examining depression by gender within each major, our study found no significant gender differences in depression levels among students in medicine, sciences, engineering, and business majors. However, females majoring in law, health sciences, and architecture exhibited significantly higher depression levels, while males showed significantly higher levels of depression in arts and letters. Contrary to our findings, a global meta-analysis conducted in 2018 reported higher depression prevalence among female medical students compared to their male counterparts (31).

Our results showed that for all tobacco products, non-medical majors had higher consumption than medical ones for both males and females. Similarly, Malaysian students showed highest percentages of tobacco consumption among non-medical fields like business and arts and social sciences (32). In contrast, current cigarette and waterpipes consumers had higher fractions of medical students than other majors in Saudi Arabia (21). In Palestine and Jordan, health related sciences had highest percentages for these products (2, 33). Concerning caffeine, our study revealed disparities between substances with health-related sciences male students being highest consumers of other types of coffee, tea and energy drinks, whereas non-medical and non-health related majors were the highest for the other substances among males. Also, females non-medical and non-health related students had higher percentages for all caffeine products than medical and health related ones. In Palestine, health sciences students consumed energy drinks and chocolate more than other majors whereas it was the non-medical ones for coffee and tea (2). In KSA, the level of caffeine intake was significantly higher in female non-health students than female health students (7). On the other hand, in UAE, no difference was seen between medical and non-medical students (8).

### 4.2 Strengths of the study

It is a comprehensive approach that studies the association between tobacco and caffeine combined with depression.

### 4.3 Limitations of the study

Our study is based on a self-reported questionnaire that may lead to recall bias. Participants may also have misreported their use of tobacco products or caffeine as well as their depressive symptoms regardless of the survey's anonymity. Additionally, bias may arise from inappropriate answers to certain sensitive questions, and from the convenient sample. It is also a cross-sectional study, where results cannot be generalized, and causal relationships cannot be deduced due to the nature of the study and the bivariate analyses conducted. Moreover, certain sociodemographic and contextual factors like income level, urban or rural status, or history of mental illness were not controlled which can influence findings. Finally, no adjustment was performed for the multiple tests used.

## 5 Conclusion

The complex interplay between substance use and mental health among university students from different majors in Lebanon, a country in socioeconomic and political instability, underscores an essential need for targeted mental health interventions, awareness campaigns, and accessible psychological support services. By recognizing the nuanced relationships between depression and the use of tobacco and caffeine, university policies can be more effectively designed to address and reduce substance-related mental health risks. Encouraging open discussions around mental health and normalizing psychological care within university environments could significantly improve student wellbeing and reduce the stigma associated with seeking support. Easily accessible counseling can be introduced in universities, educational programs and workshops can be conducted to raise awareness about depression and substances use.

## Data Availability

The raw data supporting the conclusions of this article will be made available by the authors, without undue reservation.
